# Association between malocclusion, tongue position and speech distortion in mixed-dentition schoolchildren: an epidemiological study

**DOI:** 10.1590/1678-7757-2020-1005

**Published:** 2021-08-16

**Authors:** Débora do Canto ASSAF, Jessica Klöckner KNORST, Angela Ruviaro BUSANELLO-STELLA, Vilmar Antônio FERRAZZO, Luana Cristina BERWIG, Thiago Machado ARDENGHI, Mariana MARQUEZAN

**Affiliations:** 1 Universidade Federal Santa Maria Departamento de Estomatologia Santa Maria Brasil Universidade Federal Santa Maria (UFSM), Departamento de Estomatologia, Santa Maria, Brasil.; 2 Universidade Federal Santa Maria Departamento de Fonoaudiologia Santa Maria Brasil Universidade Federal Santa Maria (UFSM), Departamento de Fonoaudiologia, Santa Maria, Brasil.; 3 Hospital de Clínicas de Porto Alegre Santa CeciliaPorto Alegre Brasil Hospital de Clínicas de Porto Alegre, Santa Cecilia, Porto Alegre, Brasil.

**Keywords:** Mixed dentition, Malocclusion, Orthodontics, Observational study, Speech-language pathologist, Speech therapy

## Abstract

**Background:**

Malocclusions are highly prevalent in childhood and adolescence, being considered a public health problem worldwide, in addition to be considered an important predictor in the tongue position and speech disorders.

**Objective:**

Evaluate the association of malocclusions with tongue position and speech distortion in mixed-dentition schoolchildren from the south of Brazil.

**Methodology:**

This cross-sectional study was performed using a database of an epidemiological survey realized in the southern of Brazil, in 2015, for evaluating the dental and myofunctional condition of the mixed-dentition from 7-13 years’ schoolchildren. The outcome variables were tongue position and speech distortion, evaluated by a trained and calibrated examiner. Characteristics regarding sociodemographic and oral health measures (Angle’s classification of the malocclusion, overjet, overbite, posterior crossbite and respiratory mode) were also assessed. Poisson regression models with adjusted robust variance were used to evaluate the association among predictors variables in the outcomes. Results are presented as prevalence ratio (PR) and 95% confidence interval (95% CI).

**Results:**

A total of 547 children were evaluated. Schoolchildren who presented anterior open bite (PR 2.36 95%CI 1.59-3.49) and having oral/oral-nasal breathing (RP 2.51 95%CI 1.70-3.71) are more likely to have altered position of the tongue. Both deep bite and being male represent protection factors for the abnormal tongue position. Regarding speech distortion, deep overbite presents a protective relationship to speech distortion (PR 0.41; 95%CI 0.24-0.71), whereas schoolchildren with posterior crossbite were more likely to present this problem (PR 1.77; 95%CI 1.09-2.88).

**Conclusion:**

Anterior open bite and posterior crossbite were the malocclusions related to speech distortion and/or altered tongue position. Oral/oral-nasal breathing was also related to myofunctional changes. Deep bite malocclusion was a protective factor for both speech problems and altered tongue position when compared to a normal overbite.

## Introduction

Malocclusions are highly prevalent in childhood and adolescence, being considered a worldwide health problem.^1^ It also can be defined as a change in growth that affects the occlusion of the teeth. In schoolchildren, malocclusion can lead to non-aesthetic traits, a poor lingual position, and, consequently, changes in speech, which can affect the quality of life.^2 , 3^ In this context, malocclusion is an important predictor to be considered in the tongue position and speech disorders.

Speech is the result of the planning and execution of sequences of movements, which require very accurate neuromuscular coordination.^3^ Articulation disorders can begin since childhood, and the prevalence is approximately 22.3% in children of a Brazilian sample.^4^ Its etiology comprises genetic factors (11%), environmental (83%) and mixed (6%).^5^ Among the factors that influence the articulatory, points’ precision are the presence and position of the teeth, mobility of the lips, cheeks, soft palate, tongue and mandible, the intraoral space for articulation and resonance of the sounds. Deviations from chewing, swallowing and breathing functions may also be associated.^6^ Moreover, the position of the anterior teeth is one of the main factors that alter the articulation of sounds, since almost 90% of all consonants are performed in this region.^7^

Previous studies have investigated the impact of different types of malocclusions on myofunctional disorders.^7 , 8 , 9^ The causal explanation between this problem and speech disorders may be related to the anatomical structure of the oral cavity, but it can also be restricted to the type of language itself.^10^ Furthermore, the relationship of malocclusion and tongue position can be explained by the Balance Theory, since teeth in the correct position help in the balance between the forces of the tongue and the labio-buccal muscles.^11^ Thus, myofunctional therapy in children with malocclusion was able to re-educate tongue positioning at rest, during swallowing and to increase the force of tongue elevation, but it was not able to improve the articulation of some phonemes,^11^ suggesting that multidisciplinary treatments, including orthodontic correction, are necessary to fully solve the problem. Between the main types of speech alterations, distortion (anterior and lateral lisp) is due to musculoskeletal, and for this reason, it is one of the interest in this study.^5^

Although there are some studies in previous literature that evaluate the relationship among malocclusions with tongue position and speech distortion,^2 , 6 , 7 , 8 , 9^ epidemiological studies with representative samples during mixed dentition using robust analysis methods are still scarce.^2 , 6 , 7 , 8 , 9^ More studies are required to prove stronger associations between tongue-positioning and speech problems with different types of malocclusions, corresponding to the real scenario. It is important for dentists and speech therapists to be aware of malocclusion and craniofacial disturbances that may interfere with the articulation of sounds and tongue position, as such problems may interfere with the interpersonal relationships of individuals throughout their lives. Thus, the study aims to evaluate the association of malocclusions with tongue position and speech distortion in mixed-dentition in schoolchildren, in the mixed dentition in the city of Santa Maria, southern Brazil. The hypothesis of the study is that altered tongue position and speech distortion are associated with malocclusion.

## Methodology

### Ethical issues

The research was approved by the Research Ethics Committee of the Federal University of Santa Maria (CEP/UFSM) under protocol number 08105512.0000.5346. The participants’ parents signed the Informed Consent Form (ICF).

### Study design and sample

This cross-sectional study was performed using database of an epidemiological survey realized in the city of Santa Maria, South Brazil, in 2015, for evaluating the dental and myofunctional condition of the mixed-dentition schoolchildren. All schools received information about the study objectives and procedures, and agreed to participate by signing the Institutional Authorization Term. In 2015, the city had an estimated population of 261,031 inhabitants, and 30,216 (11.57%) were enrolled in elementary education (Demographic Census of the Brazilian Institute of Geography and Statistics, 2015).^12^

A random sampling by double stage conglomerate was adopted. In 2014, 10,569 students were enrolled in the 26 public primary schools, where nine of these schools were randomly selected according to the size of the school and the different administrative regions. From the lists of students, 1,559 children between 7 and 13 years old were invited to participate in the study. The inclusion criteria were being in the mixed dentition phase with the first erupted upper molars. Exclusion criteria were previous or current orthodontic and/or speech therapy treatment, noticeable signs of syndromes and/or cognitive limitations. Moreover, children that had upper and lower incisors in eruption stages or had lost anterior teeth disabling the evaluation of overjet and overbite were allocated to the missing group.

The sample size calculation was performed to verify if the database sample would be enough for evaluating association between malocclusion and speech distortion, more specifically phonetic disorders. The following parameters were considered: sampling error of 5%, 95% confidence level (CI), the finite population of students enrolled in public primary schools in the city (10,569) and the prevalence of speech disorder of 23.3%.^4^ Considering a design effect of 1.2 and adding 20% for possible losses, the minimum required sample size was 375 individuals. Since this study is part of a broad epidemiological survey involving other outcomes, a larger sample than required was included.

### Calibration phase

All clinical measures were performed by four trained and calibrated dentists with orthodontics’ measures. The calibration phase was conducted in the Orthodontics laboratory, of the Dentistry course at the Federal University of Santa Maria, through a theoretical class and practical training oriented by a gold standard in the area (M.M.) The training was done by measuring different plaster models of patients from the orthodontic clinic. Posteriorly, the calibration was performed on 30 children randomly selected at the school of the data collection. They were re-evaluated twice by each dentist in a 15-day interval. The inter and intra-examiner agreement values were greater than 0.70 for all orthodontic measures (open bite, unilateral or bilateral crossbite, anterior crossbite, overjet, overbite, and Angle’s classification of malocclusion).

The speech-language assessment was performed by the same speech therapist, professor and gold standard in the area (L.C.B). To measure the intra-examiner reproducibility, 30 children were evaluated at school and reassessed after one week. The Kappa value for this assessment was upper 0.70 for all measures (positioning of the tongue, speech distortion and breathing mode).

### Orthodontic Evaluation

The children were examined by four previously trained and calibrated dentists. The examinations were performed in schools, under natural light conditions, with the child and the professional seated face to face. Data collected were: anterior open bite (present / absent), unilateral or bilateral posterior crossbite, anterior crossbite, overjet (mm), overbite (mm), and Angle’s classification of malocclusion. The World Health Organization (WHO) probe^13^ (Millennium – Golgran, SP, Brazil) was used for measurements according to a method previously described in the literature.^14^ For the overjet measurement, the patient kept his teeth closed, the probe was positioned horizontally, touching the vestibular surface of the lower central incisor and the incisal edge of upper central incisor. For the overbite measurement, the probe was used to measure how much the upper central incisor covered the crown of the lower central incisor vertically. Regarding the overjet and overbite, the measurements between 0.5 and 3.5mm were considered adequate; measurements were considered increased when ≥ 4mm and decreased when ≤ 0mm.^15^ The presence of untreated dental caries was accessed through WHO method.^13^

### Myofunctional Evaluation

The children were evaluated by a single calibrated speech therapist using an evaluation form composed of data extracted from the *Protocolo* de *Avaliação Miofuncional Orofacial com Escores* (Orofacial Myofunctional Evaluation Protocol with Scores - AMIOFE)^16^ and data taken from the *Protocolo de Avaliação Miofuncional Orofacial* (Orofacial Myofunctional Assessment Protocol – MBGR).^17^ The breathing mode of the participants was verified through a spontaneous observation of the patient, classified as nasal, mouth or oronasal according to AMIOFE. The positioning of the tongue (outcome) was evaluated during the rest and the speech, classified as normal (contained in the oral cavity) or altered (interposed to dental arches with the following subclassifications: adaptation, dysfunction or excessive protrusion) according to AMIOFE. The participant’s speech was also evaluated through automatic speech (outcome), asking the children to count from 1 to 20, telling the days of the week and the letters of the alphabet, later they were asked to describe engravings on a drawing board, so they were classified as absence or as presence of speech distortion, according to MBGR protocol. Phonetic disorders were considered those related to structural issues of the stomatognathic system, more specifically the articulatory points of fricatives and linguodentals.

### Covariables

Demographic and socioeconomic variables were collected through a structured questionnaire answered by parents at home, which included general health aspects, historical of current or previous orthodontic and/or speech therapy treatment, gender, skin color, parents schooling. Age was dichotomized by the median at <10 years and >10 years. Parents’ education was collected in complete years of formal education and later dichotomized in elementary school incomplete ( < 8 years) and complete (> 8 years).

### Statistical analysis

Data were analyzed using the STATA 14 statistical program (StataCorp, 2014. Stata Statistical Software: Release 14.1, College Station, TX: StataCorp LP). This study considered two outcomes: (1) speech distortion (absent/present) and (2) tongue position (normal/altered). It was performed a descriptive analysis of the demographic, socioeconomic and oral health measures of the sample, such as: age, gender, skin color, father and mother schooling, Angle’s classification of malocclusion (Class I, II or III), overjet (adequate, accentuated and anterior cross bite), overbite (adequate, deep bite and anterior open bite), posterior crossbite and respiratory mode, according to the distribution of outcome variables.

Unadjusted analyses were performed to provide a preliminary assessment of the association between predictor variables and outcomes. Poisson regression models with adjusted robust variance were used to evaluate the association among predictors variables in the prevalence of speech distortion and tongue position. The exploratory variables that presented a value of p≤0.20 in the univariate analysis were included in the multivariate model. Results are presented as prevalence ratio (PR) and respective 95% confidence interval (95% CI). A significance level of 0.05 was considered.

## Results

**Figure 1:**
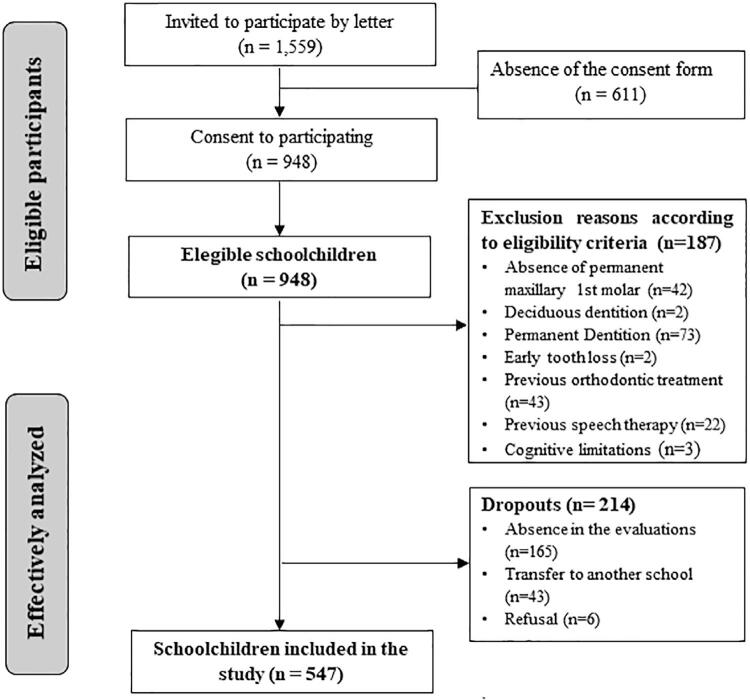
Flowchart - inclusion and exclusion criteria and sample losses

From total number of students invited to participate in the study, 948 consented and had the Consent Form signed by parents or guardians (response rate 60.8%). Of those children who agreed to participate, 547 were included in the sample. Figure 1 shows the reasons for sample losses.

Table 1 shows the sample distribution according demographic, socioeconomics characteristics, occlusal conditions and respiratory mode. Regarding the data from the occlusal evaluation, posterior crossbites were present in 11.1%, and anterior open bites in 10.1%. Adequate overbite (between 0.5 to 3.5 mm) was observed in 43.3% of the sample, while 67.8% of the sample presented adequate overjet (between 0.5 to 3.5 mm). Regarding the Angle’s classification, 85.0% were Class I, 12.8% Class II, and 2.2% Class III. The prevalence of untreated dental caries was 26.1%.

**Table 1:** Sample distribution according to demographic, socioeconomic and clinical characteristics (n=547)

Variables	N	%
**Demographic and socioeconomic variables**		
**Sex**		
Female	299	54.7
Male	248	45.3
**Age**		
7-10 years	363	66.6
11-13 years	182	33.4
**Skin color**		
White	433	79.2
Non-white	114	20.8
**Mother’s education**		
< 8 years	171	32.7
> 8 years	352	67.3
**Father’s education**		
< 8 years	203	41.7
> 8 years	284	58.3
**Clinical Variables**		
**Angle Classification**		
Class I	452	85.0
Class II	68	12.8
Class III	8	2.2
**Overjet**		
Normal	339	67.8
Anterior crossbite	21	4.2
Increased	140	2.0
**Overbite**		
Normal	222	43.3
Anterior open bite	52	10.1
Deep bite	239	46.6
**Posterior crossbite**		
Absent	478	88.9
Present	60	11.1
**Untreated dental caries**		
Absent	404	73.9
Present	143	26.1
**Respiratory mode**		
Normal	344	63.0
Oral/Oronasal	202	37.0

Values less than 547 are due to missing data.

Table 2 shows the presence or absence of the altered tongue position and speech phonetic disorders according to demographic, socioeconomics characteristics, malocclusion and respiratory mode. The percentage of children with some type of alteration in the position of the tongue was 35.8%. The prevalence of children with some type of speech distortion was found in 31.4% of the sample. The majority of children with altered tongue position and speech distortion had ages between 7 and 10 years (p>0.05).

**Table 2:** Demographic, socioeconomic and clinical variables according to speech articulation disorders and tongue position

Variables	Speech Distortion [n(%)]		Tongue position [n (%)]	
	Absent	Present	Normal	Altered
**Demographic and socioeconomic variables**				
**Sex**				
Female	252 (84.3)	47 (15.7)	237 (79.3)	62 (20.7)
Male	209 (84.6)	38 (15.4)	208 (84.9)	37 (15.1)
**Age**				
7-10 years	298 (76.0)	64 (24.0)	291 (80.6)	70 (19.4)
11-13 years	161 (88.5)	21 (11.5)	153 (84.5)	28 (15.5)
**Skin color**				
White	363 (84.0)	69 (16.0)	353 (82.1)	77 (17.9)
Non-white	98 (86.0)	16 (14.0)	92 (80.7)	22 (17.3)
**Mother’s education**				
< 8 years	144 (84.2)	27 (15.8)	141 (82.5)	30 (17.5)
> 8 years	297 (84.6)	54 (15.4)	284 (81.4)	65 (18.6)
**Father’s education**				
< 8 years	172 (84.7)	31 (15.3)	160 (78.8)	43 (21.2)
> 8 years	236 (83.4)	47 (16.6)	234 (83.3)	47 (16.7)
**Clinical Variables**				
**Angle Classification**				
Class I	379 (84.0)	72 (16.0)	371 (82.6)	78 (17.4)
Class II	59 (86.7)	9 (13.2)	53 (77.9)	15 (22.1)
Class III	10 (83.3)	2 (16.7)	8 (66.7)	4 (33.3)
**Overjet**				
Normal	284 (84.0)	54 (16.0)	290 (85.8)	48 (14.2)
Anterior crossbite	16 (76.2)	5 (23.8)	18 (85.7)	3 (14.3)
Increased	126 (90.0)	14 (10.0)	111 (80.4)	27 (19.6)
**Overbite**				
Normal	177 (80.1)	44 (19.9)	177 (80.5)	43 (19.5)
Anterior open bite	36 (69.2)	16 (30.8)	23 (44.2)	29 (55.8)
Deep bite	221 (92.5)	18 (7.5)	222 (93.3)	16 (6.7)
**Posterior crossbite**				
Absent	409 (85.7)	68 (14.3)	396 (83.4)	79 (16.6)
Present	44 (73.3)	16 (26.7)	41 (68.3)	19 (31.7)
**Untreated dental caries**				
Absent	343 (84.9)	61 (15.1)	327 (81.3)	75 (18.7)
Present	118 (83.1)	24 (16.9)	118 (83.1)	24 (16.9)
**Respiratory mode**				
Normal	292 (84.9)	52 (15.1)	306 (89.2)	37 (10.8)
Oral/Oronasal	169 (83.7)	33 (16.3)	139 (69.2)	62 (30.8)

Values less than 547 are due to missing data.

Table 3 shows the tongue position association with demographic, socioeconomics characteristics, malocclusion and respiratory mode. The presence of anterior open bite represents 2.36 times more frequency to have an alteration in the position of the tongue (PR 2.36 95%CI 1.59-3.49) in relation to the individuals with adequate overbite. Concerning being male and having the presence of a deep bite, represent protection factors to have the tongue in a normal position. Deep bite represents 61% more probability of having the tongue in normal position comparing to normal overbite (PR 0.39; 95%CI 0.23-0.67). Being male represents 35% more probability of not having an alteration in tongue position in relation to female gender (PR 0.65 95%CI 0.44-0.95). Regarding the respiratory mode, participants who have altered respiratory mode, oral or oronasal breathing, are 2.51 times more likely to have a position of the tongue altered (PR 2.51 95%CI 1.70-3.71) when compared with those with normal nasal breathing.

**Table 3:** Unadjusted and adjusted association between the independent variables for tongue position, determined using Poisson regression with robust variance

Variables	Unadjusted	P-value	Adjusted
	PRa (CIb 95%)		PRa (CIb 95%)
**Demographic and socioeconomic variables**			
**Sex**			
Female	1	0.094	1
Male	0 .72 (0.50-1.05)		0.65 (0.44-0.95)*
**Age**			
7-10 years	1	0.269	-
11-13 years	0.79 (0.53-1.19)		
**Skin color**			
White	1	0.731	-
Non-white	1.07 (0.70-1.65)		
**Mother’s education**			
< 8 years	1	0.765	-
> 8 years	1.06 (0.71-1.57)		
**Father’s education**			
< 8 years	1	0.214	-
> 8 years	0.78 (0.54-1.14)		
**Clinical Variables**			
**Angle Classification**			
Class I	1	0.340	-
Class II	1.26 (0.77-2.01)		
Class III	1.91 (0.84-4.38)		
**Overjet**			
Normal	1	0.991	-
Anterior crossbite	1.00 (0.34-2.96)		
Increased	1.37 (0.89-2.11)		
**Overbite**			
Normal	1	<0.001	1
Anterior open bite	2.85 (1.98-4.09)		2.36 (1.59-3.49)**
Deep bite	0.34 (0.19-0.59)		0.39 (0.23-0.67)**
**Posterior crossbite**			
Absent	1	<0.001	1
Present	1.90 (1.24-2.90)		1.21 (0.79-1.87)
**Untreated dental caries**			
Absent	1	0.643	-
Present	0.90 (0.59-1.37)		
**Respiratory mode**			
Normal	1	<0.001	1
Oral/Oronasal	2.85 (1.97-4.13)		2.51 (1.70-3.71)**

*P-value <0.05; **P-value <0.01; aPR, prevalence ratio; bCI, confidence interval.

Table 4 shows the speech distortion association with demographic, socioeconomics characteristics, malocclusion and respiratory mode. When the multivariate model was performed, the variables overbite and posterior crossbite showed association with speech distortion. The presence of deep bite represents 59% more prevalence of having a speech considered normal in relation to normal overbite (PR 0.41; 95%CI 0.24-0.71). Moreover, the posterior crossbite is 77% more likely to present speech distortion (PR 1.77; 95%CI 1.09-2.88) when compared with individuals without posterior crossbite.

**Table 4:** Unadjusted and adjusted association between the independent variables for speech distortion, determined using Poisson regression with robust variance

Variables	Unadjusted	P-value	Adjusted
	PRa (CIb 95%)		PRa (CIb 95%)
**Demographic and socioeconomic variables**			
**Sex**			
Female	1	0.915	-
Male	0 .97 (0.66-1.45)		
**Age**			
7-10 years	1	0.069	1
11-13 years	0.65 (0.41-1.03)		0.78 (0.48-1.27)
**Skin color**			
White	1	0.615	-
Non-white	0.87 (0.53-1.45)		
**Mother’s education**			
< 8 years	1	0.905	-
> 8 years	0.97 (0.63-1.48)		
**Father’s education**			
< 8 years	1	0.693	-
> 8 years	1.08 (0.71-1.64)		
**Clinical Variables**			
**Angle Classification**			-
Class I	1	0.569	
Class II	0.82 (0.43-1.58)		
Class III	1.04 (0.28-3.76)		
**Overjet**			
Normal	1	0.098	1
Anterior crossbite	1.49 (0.66-3.33)		0.79 (0.33-1.91)
Increased	0.62 (0.35- 1.08)		0.69 (0.39-1.23)
**Overbite**			
Normal	1	<0.001	1
Anterior open bite	1.54 (0.95-2.51)		1.63 (0.86-3.11)
Deep bite	0.37 (0.22-0.63)		0.41 (0.24-0.71)**
**Posterior crossbite**			
Absent	1	0.010	1
Present	1.87 (1.16-3.00)		1.77 (1.09-2.88)*
**Untreated dental caries**			
Absent	1	0.609	-
Present	1.11 (0.72-1.72)		
**Respiratory mode**			
Normal	1	0.704	-
Oral/Oronasal	1.08 (0.72-1.61)		

*P-value <0.05; **P-value <0.01; aPR, prevalence ratio; bCI, confidence interval.

## Discussion

Our findings showed that malocclusions are related to altered tongue position and speech distortion, in accordance with our conceptual hypothesis. Regarding tongue position, schoolchildren that presented anterior open bite and oral/oral-nasal respiratory are more likely to have altered positions of the tongue. Considering speech distortion, posterior crossbite was also associated whit it. It was observed that 35.8% of the children presented altered tongue position, and 31.4% had speech distortion. The prevalence of speech disorders in previous studies ranged from 13^18^ to 42%^4^ and around 40%^19^ to altered tongue position, so the findings of this study agrees with the literature.

Most of children with speech distortion and language impairment were younger than 10 years, but this result did not present a statistically significant difference, agreeing with the study of Rabelo, et al.^4^ (2011), where there was no association between age and articulation disorders, except for children under 5 years of age. Furthermore, there was no association between gender and articulation disorders, in agreement with studies by Rabelo, et al.^4^ (2011) and Goulart and Chiari^18^ (2007). However, the male gender presented a protective factor for altered tongue position. A previous study showed that sex may affect palatal morphology among individuals and consequently, the tongue position,^20^ as well as found in the present study.

A positive association was found between the malocclusion of posterior crossbite and speech distortion. This result agrees with the findings of Farret, et al.^21^ (1998) about a strong association between posterior crossbite and speech alterations in mixed and deciduous children dentition. The clinician must be aware that the presence of posterior crossbite was the only malocclusion in this study that represents a risk factor for both altered tongue position and speech distortion in children. The posterior crossbite is not self-corrected during the development of the dentition and its early interception is indicated to allow adequate growth of the craniofacial structures.^22^ Furthermore, the results of this study also show the need for early correction to avoid functional speech impairment and tongue posture.

Deep overbite appeared as a protective factor for altered tongue position and speech distortion. Previous studies do not corroborate this result. Whereas Laine, Linnasalo and Jaroma^23^ (1987) and Leavy^7^ (2016) found no association between these variables, Farronato, et al.^24^ (2012) and Lubit^25^ (1967) found an association between the deep bite and speech distortion. The main differences may be due to methodological, regional and age aspects among the studies. Our findings suggest caution in indicating the treatment of mild to moderate deep bites in young patients in mixed dentition. However, severe deep bites and other associated complications should be indicated for treatment.

Malocclusions of Class I, II and III presented no association with phonetic disorders. This disagrees with Farronato, et al.^24^ (2012), who stated that Class II relationship was considered a low risk but the Class III relationship was considered as a high risk for articulation disorders. This difference can be explained by the high adaptability of Angle Class II individuals that were able to adjust their joints to produce all vowels.^26^ Moreover, the anterior open bite was associated to altered tongue position; however, it was not associated with speech distortion, agreeing with a previous study,^27^ which evaluated children aged between 3 to 7 years with anterior open bite using a multivariate analysis. Other studies, on the other hand, have found association between open bite and speech distortion^28 - 30^ , which may be explained due to differences in methodological and population aspects among the studies. Since our study was conducted using a representative sample of the population of the city of Santa Maria, we hypothesized that, in this general population, many children with anterior open bite might be adapted to their anatomical condition and might be able to establish a normal speech function. This adaptive capacity must be related to the severity of the open bite. However, in this study, for statistical purposes, it was not possible to stratification the open bite cases into mild, moderate, and severe because of the low prevalence of this malocclusion (which was about 10% - 51 children). Another difference in the present study was the children’s native language (Portuguese). It is known that different languages require the production of different phonemes and it can influence research results.

The respiratory mode was associated with changes in tongue position, but not with changes in speech, partially agreeing with Hitos, et al.^6^ (2013), who found the two types of changes present in mouth breathers. In fact, changes at the structural level, such as position and mobility of the soft and rigid structures of the stomatognathic system and intraoral space, can suffer more easily from the respiratory mode^5^ compared to the influence on other functional patterns. Furthermore, changes at the functional level, such as the articulation points of the speech, can adapt more easily when atypical appear in other functions.^6 , 7^ These findings also suggest the importance of a multidisciplinary approach. The association found between breathing mode and tongue position can contribute to the evaluation of patients that are consulting with different professionals such as dentists, otolaryngologist, and speech therapists. When a functional imbalance is observed, the professional should treat or refer the patient to a multi-professional treatment.

The study has some strong points, such as the high sample size and epidemiologic nature, combined with complete dental and myofunctional evaluations have the potential to clarify doubts about the associations between malocclusion and tongue position, speech distortion, and breathing mode, enabling dentists and speech therapists to plan interdisciplinary treatments, guaranteeing an adequate occlusal relationship and phono-articulatory function. Another strength of this study is that children were in mixed dentition, since there are few epidemiological surveys evaluating schoolchildren in this phase. As a limitation of this study, a cross-sectional evaluation was performed, which cannot establish a cause-effect relationship between malocclusions, tongue position, speech distortion, and respiratory mode. Furthers longitudinal studies are suggested on this issue. Furthermore, the different types of posterior crossbite (unilateral, bilateral and functional) and severity of the anterior open bite (severe, moderate and mild) were not considered in this study, which might limit our findings. It can be assumed that, the more specific the diagnosis, the better the interpretation and understanding of the associations will be. Thus, further studies are suggested to assess the association between tongue position, speech distortion and the aforementioned subtypes of malocclusions.

In conclusion, the posterior crossbite was associated with altered tongue position and speech distortion. Anterior open bites and oral/oronasal breathing mode were also associated with altered tongue position, but do not interfere in speech distortion. Deep bite appears as a protection factor for speech distortion and altered tongue position. These findings reinforce the importance of a multi-professional approach for treating children who have different types of malocclusion.
